# Biological implications and therapeutic potential of phosphodiesterase inhibitors: A review

**DOI:** 10.1097/MD.0000000000047683

**Published:** 2026-03-06

**Authors:** Mansour Alnazari, Abdulaziz Bakhsh, Sulaiman Abdullah, Saeed Al Qahtani, Walaa Borhan, Emad Rajih, Ahmed M. Alshehri

**Affiliations:** a Department of General and Specialized Surgery, College of Medicine, Taibah University, Madinah, Saudi Arabia; b Department of Basic Medical Sciences, College of Medicine, Taibah University, Madinah, Saudi Arabia; c Department of Surgery, King Faisal Specialist Hospital & Research Center, Madinah, Saudi Arabia; d Department of Clinical Pharmacy, College of Pharmacy, Prince Sattam bin Abdulaziz University, Al-Kharj, Riyadh, Saudi Arabia.

**Keywords:** cyclic adenosine monophosphate, cyclic guanosine monophosphate, phosphodiesterase 4, phosphodiesterase 5, phosphodiesterase inhibitors, therapeutic applications

## Abstract

Phosphodiesterase (PDE) inhibitors regulate cyclic adenosine monophosphate and cyclic guanosine monophosphate pathways, which influence neurodevelopment, cardiovascular function, and immune responses. Multiple PDE families exist, classified as dual-substrate (PDE1, PDE2, PDE3, PDE10, PDE11) or non-dual-substrate (PDE4, PDE5, PDE6, PDE7, PDE8, PDE9), each with distinct biological roles. This review summarizes the therapeutic applications of PDE inhibitors, evaluates evidence across different disease domains, and highlights challenges and future research priorities. A narrative review of published studies and clinical trial data was conducted, focusing on pharmacological properties, therapeutic relevance, and safety profiles of PDE inhibitors. Sources included PubMed, ClinicalTrials.gov, and regulatory reports. Dual-substrate PDEs demonstrate therapeutic potential in Alzheimer disease (PDE1), anxiety and memory enhancement (PDE2), and heart failure (PDE3), although chronic PDE3 inhibition may increase risks. Non-dual-substrate PDEs, such as PDE4 and PDE5, are clinically established for asthma, chronic obstructive pulmonary disease, psoriasis, erectile dysfunction, and pulmonary hypertension. Advances in structure–activity relationship studies have produced more selective and potent inhibitors. However, adverse effects, such as nausea (PDE4 inhibitors) and cardiovascular risks (long-term PDE3 inhibitors), remain limiting factors. PDE inhibitors represent a rapidly evolving therapeutic class with broad clinical applications. Their further development requires strategies to minimize adverse effects, improve selectivity, and better define disease-specific roles. Future research should focus on precision medicine approaches to fully harness their therapeutic potential.

## 1. Introduction

The cyclic nucleotide signal transduction system, which includes the 3′ to 5′ (nucleotide orientation) cyclic adenosine and guanosine monophosphate (cyclic adenosine monophosphate [cAMP] and cyclic guanosine monophosphate [cGMP]) signaling systems, has significant biological implications and associated therapeutic potential, thus generating considerable research interest. Both cAMP and cGMP serve as second messengers in the nonprotein signaling system, crucial for relaying signals produced by cell surface receptor–ligand interactions to effector proteins.^[1,2]^ Effector proteins represent a variety of proteins, including cAMP- and cGMP-activated protein kinases. Therefore, they play a role in several biological responses.^[3]^

Sophisticated homeostatic mechanisms involving adenylyl and guanylyl cyclases and cyclic nucleotide phosphodiesterase (PDE) maintain intracellular messenger concentrations. Adenylyl cyclase plays a significant role in the conversion of adenosine monophosphate/guanosine monophosphate into 3′ to 5′ (nucleotide orientation)-cAMP/cGMP through its activation secondary to receptor–ligand interaction, while guanylyl cyclase inhibits the cyclic nucleotide deactivation process.^[1]^ Throughout the 20th century, the therapeutic potential of PDEs has been illustrated,^[4,5]^ leading to continuous advancement in this field with different clinical indications. As of 2022, ClinicalTrials.gov listed over 1800 clinical trials investigating PDE inhibitors. However, despite this substantial research activity, only a limited number of PDE inhibitors have received regulatory approval, reflecting the challenges in translating promising experimental findings into safe and effective clinical therapies.^[2]^

**Figure 1.**
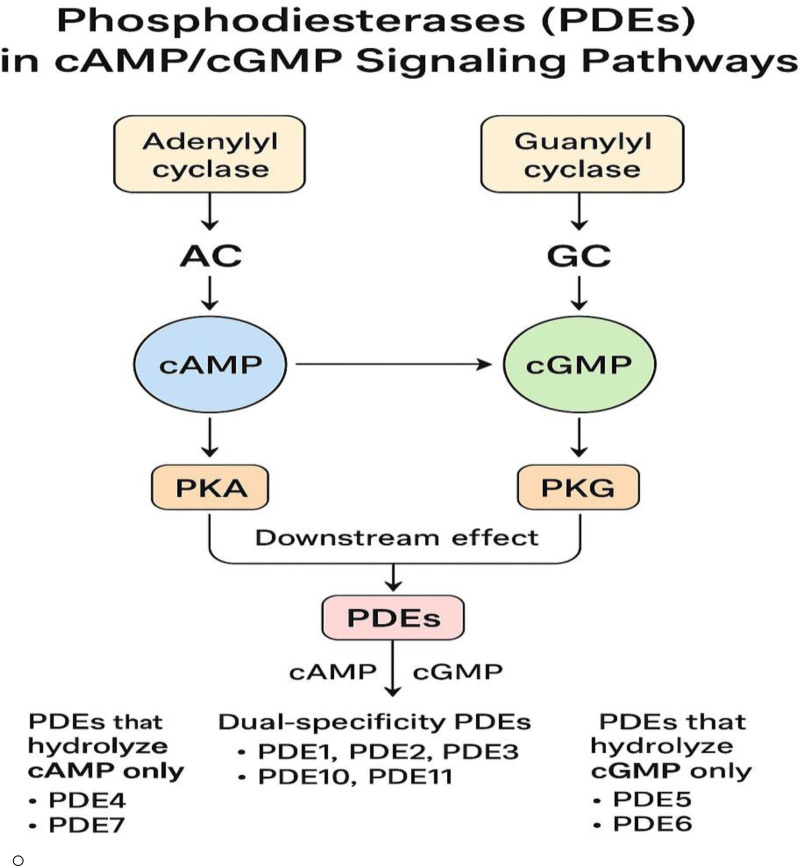
Phosphodiesterases in cyclic adenosine monophosphate and cyclic guanosine monophosphate signaling pathways. cAMP = cyclic adenosine monophosphate, cGMP = cyclic guanosine monophosphate, PDE = phosphodiesterase, PKA = protein kinase A, PKG = protein kinase G.

**Figure 2.**
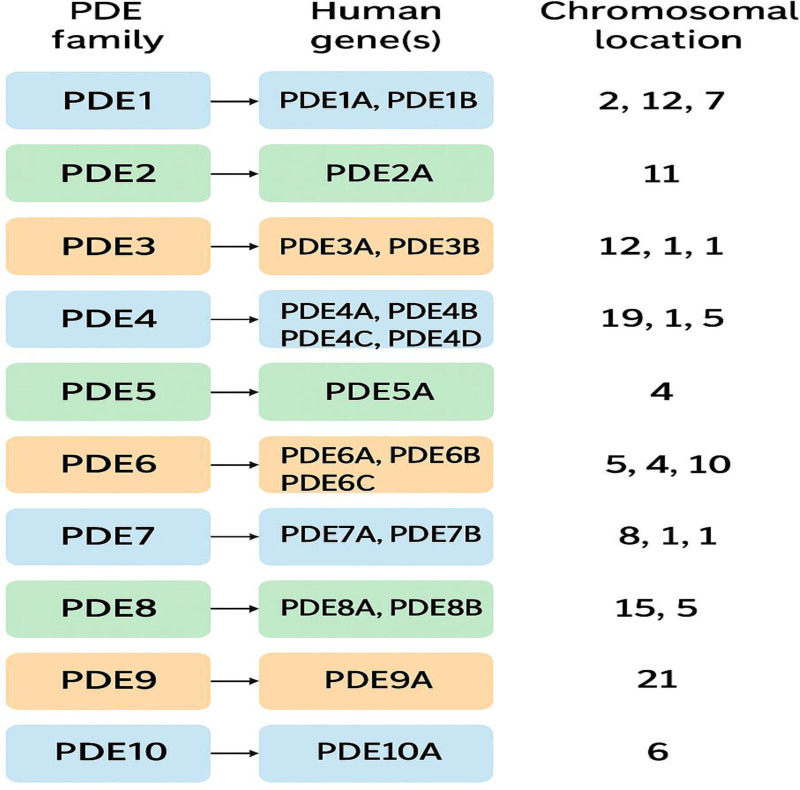
Flow diagram of human PDE families, genes, and chromosomal locations. PDE = phosphodiesterase.

The PDE superfamily encompasses 11 families encoded by 21 genes (Fig. 1) responsible for catalyzing 3′-cyclic phosphate bond hydrolysis in cyclic nucleotide molecules.^[6]^ The family designation is based on enzyme structure, particularly the homology of the C-terminal catalytic domain, which binds with the N-terminal regions to form the enzyme structure.^[6]^ Each family is functionally unique in its specifications toward cyclic nucleotide substrates (Fig. 2). Dual-substrate PDEs (PDE1-PDE3, PDE10, and PDE11) can hydrolyze cAMP and cGMP with similar affinity, whereas PDEs specific to cAMP, such as PDE4, PDE7, and PDE8, have a higher affinity for cAMP, and cGMP-specific ones, such as PDE5, PDE6, and PDE9, are more selective toward cGMP.^[6,7]^

PDE inhibitors act on specific PDEs in specific cells and have been approved for managing chronic obstructive pulmonary disease (COPD), pulmonary hypertension, psoriasis, psoriatic arthritis, and atopic dermatitis.^[8]^ Table 1 shows the clinical relevance of PDE families. PDE activity inhibition increases cAMP and cGMP levels. Therefore, several therapeutic effects can be achieved, including smooth muscle relaxation, increased blood flow, and inflammatory response inhibition.^[4]^

**Table 1:** Phosphodiesterase (PDE) families (PDE1–PDE11): substrate preference, major isoforms, physiological roles, and clinical relevance.

PDE family	Substrate preference	Isoforms/notes on splicing	Major physiological roles	Clinical relevance/diseases and drugs
PDE1	Dual-specificity (cAMP and cGMP; Ca^2+^/calmodulin-regulated)	Multiple isoforms produced by alternative splicing and promoter use	Integrates Ca^2+^ signaling with cyclic-nucleotide signaling (cardiac, neuronal, vascular smooth muscle)	Implicated in cardiac hypertrophy, memory/behavioral processes; potential target for neuro/cardiac disease research.
PDE2	Dual (hydrolyzes both cAMP and cGMP; cGMP can allosterically regulate activity)	Several splice variants reported	Regulates cross-talk between cGMP and cAMP pools (heart, brain, adrenal)	Cardiac and neuronal signaling; studied for heart failure and cognitive function modulation.
PDE3	Dual (hydrolyzes both; high affinity for cAMP with cGMP as competitive inhibitor)	Isoforms differ in N-terminal targeting motifs	Key regulator of cardiac contractility, platelet aggregation, lipolysis	PDE3 inhibitors (cilostazol, milrinone) used clinically (intermittent claudication, acute heart failure/positive inotrope); dysregulation linked to heart disease.
PDE4	cAMP-specific	Large family of isoforms (extensive alternative promoters/splicing)	Major regulator of cAMP signaling in immune cells, lung, brain, heart	PDE4 inhibitors (roflumilast, apremilast, crisaborole) used in COPD, psoriasis, atopic dermatitis; side effects (nausea) and wide clinical interest.
PDE5	cGMP-specific	Multiple splice variants	Vascular smooth muscle relaxation via cGMP (NP–GC–cGMP pathway)	PDE5 inhibitors (sildenafil, tadalafil) – erectile dysfunction, pulmonary arterial hypertension; ocular effects reported (retina), and important drug interactions.
PDE6	cGMP-specific (photoreceptor PDE)	Photoreceptor-specific subunits and regulatory subunits (gamma subunits exist)	Central to phototransduction in rod/cone cells	Mutations cause retinitis pigmentosa/photoreceptor degeneration; key in retinal disease research.
PDE7	cAMP-specific	A small number of isoforms	Immune cell cAMP control; expressed in brain and immune tissues	Investigational target for inflammatory and neuropsychiatric disorders.
PDE8	cAMP-specific	Isoforms exist	Regulates cAMP in steroidogenic tissues (Leydig, adrenal), thyroid axis	*PDE8B* mutations associated with adrenal hyperplasia and endocrine phenotypes; potential role in steroidogenesis.
PDE9	cGMP-preferring	Isoforms from alternative splicing	cGMP regulation in brain, kidney, and vasculature	Investigated for cardiovascular and cognitive disease; selective PDE9 inhibitors in research.
PDE10	Dual (hydrolyzes both; strong activity toward cAMP in striatum)	Brain-enriched isoforms	Highly expressed in striatum; regulates dopaminergic signaling and motor control	Target for schizophrenia and Huntington disease research; *PDE10A* PET is used as a biomarker in neuro studies.
PDE11	Dual (hydrolyzes both cAMP and cGMP)	Multiple splice variants	Expressed in adrenal, prostate, brain; modulates cyclic-nucleotide signaling	Mutations/variants linked to adrenal nodular disease and other endocrine phenotypes; emerging interest in tumor biology.

cAMP = cyclic adenosine monophosphate, cGMP = cyclic guanosine monophosphate, COPD = chronic obstructive pulmonary disease, NP–GC–cGMP = natriuretic peptide–guanylyl cyclase–cyclic guanosine monophosphate, PDE = phosphodiesterase, PET = positron emission tomography.

This review aims to provide a comprehensive understanding of the current knowledge on PDE inhibitors, with a focus on dual-substrate PDEs, including their design, synthesis, and structure–activity relationship (SAR) studies.

### 1.1. Dual-substrate PDEs

Dual-substrate PDEs, including PDE1, PDE2, PDE3, PDE10, and PDE11, can break down multiple cyclic nucleotide types, such as cAMP and cGMP, into inactive products. These enzymes possess dual specificity, meaning they can act on cAMP and cGMP with varying affinities depending on the isoform (Table 1).^[6]^ Dual-substrate PDEs play a key role in regulating the levels of cyclic nucleotides in cells, and their activity can affect various cellular processes, such as neurodevelopment, neuroplasticity, and cognitive functions.^[7]^

PDE1 can break down cAMP and is key in regulating cognitive function in specific brain areas.^[9]^ It has received significant attention as a potential therapeutic target in Alzheimer disease (AD) due to its role in maintaining cAMP and cGMP balance in the brain.^[10]^ PDE1 comprises 3 subtypes: *PDE1A*, *PDE1B*, and *PDE1C*, each with different tissue distribution patterns and functions. *PDE1B*, in particular, is responsible for over 90% of the brain activity associated with learning and memory, making it a promising target for treating neurodevelopmental disorders.^[11]^ While PDE1 and its subtypes convert cAMP and cGMP into inactive byproducts, *PDE1B* is more inclined to break down cGMP. However, the lack of specific data on the functional implications of these PDE1 isoforms and the challenges in creating selective inhibitors have hindered progress in understanding the precise role of each PDE1 isoform.^[12]^ Recent studies have also linked *PDE1* gene expression to cognitive behavior in specific populations, emphasizing the need to further examine PDE1 through pharmacological means.^[13]^ In addition, the cAMP/protein kinase A (PKA)/cAMP response element-binding protein (CREB)/brain-derived neurotrophic factor (BDNF) and cGMP/protein kinase G (PKG)/CREB/BDNF pathways are essential for neuroplasticity and memory function; however, PDE1 can hinder these processes by breaking down cAMP/cGMP and disrupting cognitive function.^[9,14]^ Nevertheless, inhibiting high PDE1 expression in the human brain can protect against neurodegeneration.^[15]^

PDE2 regulates the activity of cyclic nucleotides such as cAMP and cGMP, which transmit signals between neuronal cells. PDE2 inhibition increases PKA and PKG levels, ultimately resulting in the phosphorylation of CREB, which, in turn, regulates the transcription of genes such as BDNF.^[16]^ According to Scott Bitner,^[17]^ BDNF controls the expression of genes involved in synaptic plasticity and neuron survival, including postsynaptic density protein 95 and synaptophysin. Specifically, postsynaptic density protein 95 crucially links neuronal nitric oxide synthase to the *N*-methyl-D-aspartate receptor signaling complex, activating a downstream cGMP-dependent neuroprotective pathway, which regulates the progression of various mental disorders.^[18]^ Recent studies indicate that PDE2 is overexpressed in various neurological conditions, including depression and anxiety.^[16,19]^ Increasing evidence suggests that PDE2 is present in areas of the brain associated with stress-related mental disorders and cognitive dysfunction.^[20]^ In addition, research has shown that blocking PDE2 can enhance memory and cognitive function in models of AD. Furthermore, specific PDE2 inhibitors, such as Bay 60–7550, ND7001, and EHNA, have been found to have antidepressant, anxiolytic, and memory enhancement effects.^[21]^ These findings indicate that PDE2 plays a significant role in post-traumatic stress disorder-like dysfunction, wherein the PDE2 inhibitor Bay 60–7550 regulates pre- and postsynaptic proteins and PKA/PKG-dependent pathways.^[21]^

The PDE3 and PDE4 isoforms have traditionally been considered the primary PDEs for regulating cardiac contractility in mice and other species.^[22]^ Studies have shown that both PDE3 and PDE4 activity regulate baseline calcium transients and myocardial contractility by modulating cAMP/PKA-dependent sarcoplasmic reticulum (SR) calcium-ATPase (sarcoplasmic/endoplasmic reticulum Ca^2+^-ATPase isoform 2a [SERCA2a] pump) activity in microdomains that do not contain ryanodine receptors or L-type calcium channels.^[23–25]^ In humans, PDE3 inhibitors have been shown to enhance myocardial contractility, relaxation, and diastolic function,^[26,27]^ which have been linked to beta-receptor- and cAMP/PKA-dependent increases in SR calcium uptake^[27]^ via the PKA-dependent phosphorylation of phospholamban (PLN).^[28]^ However, beta-receptor-dependent effects on L-type calcium channels have also been reported in rats.^[29]^ These observations support a model in which PDE3 enzymes regulate cAMP compartmentalization and calcium transients within microdomains containing SERCA2a as well as other regulatory proteins.^[23–25]^ Despite their short-term benefits, chronic PDE3 inhibitor administration increases mortality.^[30,31]^ Despite these side effects, PDE3 inhibitors are still used to treat peripheral vascular disease.^[32]^ The specific PDE3 isoform that regulates myocardial contractility remains unclear, with different studies suggesting that it is either *PDE3A*^[33]^ or *PDE3B*.^[34]^ The *PDE3A*^−^/^−^ and *PDE3B*^−^/^−^ mice were used to better understand PDE inhibitor isoform dependence for regulating myocardial contractility. The findings established that *PDE3A*, not *PDE3B*, regulates baseline contractility in the murine myocardium via the cAMP-dependent modulation of calcium transients, SR calcium-ATPase activity, and PLN phosphorylation in SERCA2a-containing SR microdomains of ventricular cardiomyocytes.

Zhang et al^[35]^ investigated the SAR of PDE10 inhibitors and their potential as anticancer therapeutics. They used computational methods to design and synthesize these inhibitors and then evaluated their inhibitory activity against PDE10 and their cytotoxicity against cancer cells. The results showed that PDE10 inhibitors displayed potent inhibitory activity against PDE10 and exhibited cytotoxicity against cancerous cells. Therefore, the authors concluded that PDE10 inhibitors could be effective anticancer agents. *PDE10A* is a highly concentrated enzyme in specific brain regions, specifically the putamen and caudate nucleus. It plays a crucial role in controlling the location, duration, and intensity of cyclic nucleotide signaling in these areas.^[36]^ Therefore, targeting *PDE10A* has been identified as a potential strategy for developing effective treatments for various disorders, including schizophrenia, psychosis, Huntington disease, and other conditions associated with the central nervous system.^[37]^

According to Hannah-Shmouni et al^[38]^ and Rall and Sutherland,^[39]^ *PDE11A* can break down both cAMP and cGMP. Butcher and Sutherland^[40]^ and Liu et al^[41]^ indicate that this enzyme comprises 4 different splice variants (*PDE11A1*, *PDE11A2*, *PDE11A3*, and *PDE11A4*) that vary in terms of where they are expressed in the body and the structure of their N-terminal regulatory regions. Hetman et al^[42]^ and Fawcett et al^[43]^ argued that the N-terminal domain plays a regulatory role while the C-terminal domain is catalytic. Yuasa et al^[44]^ and Yuasa et al^[45]^ reported that the longest *PDE11A* isoforms found in mice and humans have a protein sequence homology of approximately 95%. Among the different splice variants, *PDE11A* is most highly expressed in the prostate.^[43,44]^ Hetman et al^[42]^ and Fawcett et al^[43]^ further indicated that *PDE11A1* and *PDE11A3* are found in the spleen and *PDE11A4* in the hippocampus. *PDE11A* is also present in the liver, skeletal muscle, pituitary gland, pancreas, and kidneys.^[43,44]^ Therefore, the expression and structure of *PDE11A* vary depending on tissue location. The function of different PDE isoforms varies across different human tissues, with *PDE11A* having 4 splice variants.^[43,44]^ *PDE11A* expression is most prevalent in the prostate but can also be present in the liver, pancreas, spleen, skeletal muscle, pituitary, hippocampus, and kidneys. *PDE11A* regulates both cAMP and cGMP levels^[38]^ and is present in many cancer cells, with several mutations identified. Three *PDE11A* truncations and 2 substitution mutations were discovered in 745 patients.^[46,47]^

### 1.2. Non-dual-substrate PDEs

Non-dual-substrate PDEs exclusively target either cAMP or cGMP. PDEs that are specific to cAMP, such as PDE4, PDE7, and PDE8, have a greater affinity for cAMP, whereas those specific to cGMP, such as PDE5, PDE6, and PDE9, have a greater specificity for cGMP.^[6,7]^

PDE4, comprising *PDE4A*, *PDE4B*, *PDE4C*, and *PDE4D* subtypes, is found in the heart and small intestine, immune cells, and the brain.^[48,49]^ Unlike other PDEs, PDE4 has a higher affinity for cAMP than cGMP and regulates brain function, immune cell activation, cardiovascular functions, and fertility.^[48,49]^ PDE5, consisting of subtypes *PDE5A1–3*, localizes in the lung, penis, smooth muscle, platelets, brain, and cardiac muscle and regulates the activity of cGMP.^[48,49]^ PDE6, made up of subtypes *PDE6A*, *PDE6B*, and *PDE6C*, is found in photoreceptors and the pineal gland and plays a role in regulating cGMP levels in these cells.^[48,49]^ PDE7, comprising subtypes *PDE7A1–2* and *PDE7B1–3*, is found in immune cells, skeletal and cardiac muscles, and the brain and plays a role in regulating cAMP activity.^[48,49]^ Consisting of subtypes *PDE8A1–5* and *PDE8B1–3*, PDE8 localizes in immune cells, the heart, ovary and testes, thyroid gland, placenta, brain, and adrenal glands, and is involved in regulating cAMP activity and hormone levels.^[48,49]^ PDE9, which includes subtypes *PDE9A1–6*, is present in the kidney, spleen, gut, and prostate and regulates cGMP activity in relation to energy balance.^[48,49]^ PDE10, composed of subtypes *PDE10A1–2*, is located in the brain, testis, and thyroid, where it regulates cGMP activity.^[48,49]^

## 2. Discussion

The cyclic nucleotide PDE superfamily is a diverse group of enzymes that regulate the intracellular concentrations of second messengers, such as cAMP and cGMP. PDEs catalyze the hydrolysis of the 3′-cyclic phosphate bond in cyclic nucleotide molecules, resulting in inactive compounds.^[6]^ PDE inhibitors, which target specific PDEs, have been studied extensively for their therapeutic potential against various indications, including COPD, pulmonary arterial hypertension, psoriasis, psoriatic arthritis, atopic dermatitis, and erectile dysfunction.^[8]^

The PDE superfamily comprises 11 families encoded by 21 genes, each with unique specificities toward cyclic nucleotide substrates. On the one hand, the dual-substrate PDEs, including PDE1–PDE3, PDE10, and PDE11, catabolize cAMP and cGMP.^[6]^ On the other hand, cAMP-specific PDEs such as PDE4, PDE7, and PDE8 exhibit higher affinities for cAMP, while cGMP-specific PDEs such as PDE5, PDE6, and PDE9 are more specific for cGMP.^[6,7]^

The synthesis of PDE inhibitors has been intensively researched for several decades, beginning in the 1960s when the first PDE inhibitor, “dipyridamole,” was discovered.^[4]^ Since then, various strategies have been employed to design and synthesize PDE inhibitors, including rational design, high-throughput screening, and combinatorial chemistry.^[2]^ The rational design approach involves identifying key structural features of PDEs essential for their activity and the subsequent design of inhibitors that target these features. High-throughput screening, on the other hand, involves screening large libraries of compounds for their ability to inhibit PDEs. Combinatorial chemistry, a more recent approach, involves constructing large libraries of compounds by combining small building blocks.

Studies into PDE inhibitor SARs have yielded valuable insights into the molecular mechanisms underlying PDE inhibition. These studies have shown that the binding of PDE inhibitors to the active sites of PDEs is mediated by hydrogen bonding, van der Waals interactions, and hydrophobic interactions.^[2]^ Furthermore, SAR studies have revealed that PDE inhibitor binding is highly dependent on the size and shape of the inhibitor, as well as the position and orientation of functional groups.^[2]^

PDE1 breaks down cAMP and plays a key role in regulating cognitive functions in certain brain areas. PDE1 has been believed to have potential therapeutic benefits in AD due to its role in maintaining the balance of cAMP and cGMP in the brain. PDE1 consists of 3 subtypes – *PDE1A*, *PDE1B*, and *PDE1C* – each with different tissue distribution patterns and functions. Among these, *PDE1B* is responsible for >90% of brain activity related to learning and memory, making it a promising target for treating neurodevelopmental disorders. However, the lack of specific data on the functional implications of these PDE1 isoforms and the challenges in developing selective inhibitors have hindered progress in understanding the precise role of each PDE1 isoform.

Inhibiting PDE2 increases PKA and PKG, ultimately resulting in the phosphorylation of CREB, which regulates genes, such as BDNF. Recent studies indicate that PDE2 is overexpressed in various neurological conditions, including depression and anxiety. Blocking PDE2 can enhance memory and cognitive function in models of AD. Furthermore, certain PDE2 inhibitors, such as Bay 60–7550, ND7001, and EHNA, have been found to have antidepressant, anxiolytic, and memory enhancement effects.

Historically, PDE3 and PDE4 isoforms have been recognized as the main PDEs responsible for regulating cardiac contractility in mice and other species. Studies have shown that their activity regulates baseline calcium transients and myocardial contractility by modulating SERCA2a pump activity in microdomains that do not contain ryanodine receptors or L-type calcium channels. In humans, PDE3 inhibitors enhance myocardial contractility, relaxation, and diastolic function, which have been linked to beta-receptor- and cAMP/PKA-dependent increases in SR calcium uptake via PKA-dependent phosphorylation of PLN.

Among PDE inhibitors, non-dual-substrate PDEs have been the focus of much research due to their exclusive targeting of either cAMP or cGMP. These enzymes, including PDE4, PDE5, PDE6, PDE7, PDE8, PDE9, and PDE10, have different subtypes found in various tissues and cells throughout the body. For example, PDE4, comprising subtypes *PDE4A*, *PDE4B*, *PDE4C*, and *PDE4D*, has a higher affinity for cAMP than for cGMP and plays a role in regulating brain function, immune cell activation, cardiovascular functions, and fertility.^[48,49]^ PDE5, consisting of subtypes *PDE5A1–3*, is found in the lung, penis, smooth muscle, platelets, brain, and cardiac muscle and regulates cGMP activity.^[48,49]^ PDE6, made up of subtypes *PDE6A*, *PDE6B*, and *PDE6C*, is found in photoreceptors and the pineal gland and plays a role in regulating cGMP levels in these cells.^[48,49]^ PDE7, comprising subtypes *PDE7A1–2* and *PDE7B1–3*, is found in immune cells, skeletal and cardiac muscles, and the brain and is involved in regulating cAMP activity.^[48,49]^ Comprising subtypes *PDE8A1–5* and *PDE8B1–3*, PDE8 is present in immune cells, the heart, ovary and testes, thyroid gland, placenta, brain, and adrenal glands. It is involved in regulating cAMP activity and hormone levels.^[48,49]^ PDE9, consisting of subtypes *PDE9A1–6*, localizes in the kidney, spleen, gut, and prostate and regulates cGMP activity associated with energy balance.^[48,49]^ PDE10, made up of subtypes *PDE10A1–2*, is found in the brain, testis, and thyroid, where it regulates cGMP activity.^[48,49]^

Targeting specific PDEs with inhibitors has shown potential in treating various conditions, such as asthma, erectile dysfunction, and heart failure.^[6,7]^ However, due to potential side effects and drug interactions, further research is necessary to fully comprehend the clinical implications of targeting non-dual-substrate PDEs with inhibitors.

## 3. Limitations

In this review, we discussed the significance of the cyclic nucleotide signal transduction system in relaying signals produced via receptor–ligand interactions at the cell surface to effector proteins. We also highlighted the role of PDEs in maintaining the intracellular concentrations of second messengers within the cyclic nucleotide system. However, it is essential to note that while the potential of PDEs as a drug development target has been recognized for several decades, only a relatively small number of these agents have entered the market, while several initially promising therapeutic agents have yet to prove successful. Additionally, the diversity of the PDE superfamily, comprising 11 families encoded by 21 genes and responsible for catalyzing 3′-cyclic phosphate bond hydrolysis in cyclic nucleotide molecules, highlights the complexity of intracellular signaling pathways and intricate interactions between different PDEs.

## 4. Recommendations

Despite the limitations, PDE inhibitors remain a promising drug development target for various clinical indications. Further research should focus on characterizing individual PDE families, including their specificities toward cyclic nucleotide substrates and their roles in regulating various physiological processes. Identifying specific PDE subtypes and their locations in the body can also lead to the development of more targeted and effective PDE inhibitors. Additionally, understanding the intricate interactions between different PDEs can lead to the development of combination therapies targeting multiple PDEs, potentially leading to more effective treatment outcomes.

## 5. Conclusion

Here, the significance of the cyclic nucleotide signal transduction system, specifically the cAMP and cGMP signaling systems, was discussed, highlighting the role of PDEs in maintaining the intracellular concentrations of second messengers within the cyclic nucleotide system. The potential of PDEs as drug development targets was recognized, as evidenced by several clinical studies involving PDE inhibitors. However, only a small number of these agents have entered the market. The diversity of the PDE superfamily was also discussed, with each family having distinct properties and specificities toward cyclic nucleotide substrates. Several PDE inhibitors, which act on specific PDEs in target cells, have been approved by the U.S. Food and Drug Administration for several conditions, including COPD, erectile dysfunction, pulmonary arterial hypertension, and inflammatory conditions, such as psoriasis and atopic dermatitis. The role and function of several PDE enzymes, including PDE1, PDE2, PDE3, PDE10, and PDE11, were described, with each enzyme having specific roles in regulating cellular processes.

PDE inhibitors have been the subject of intense research for their therapeutic potential in various indications. The design, synthesis, and SAR studies of PDE inhibitors have provided valuable insights into the molecular mechanisms of PDE inhibition and led to the development of several clinically useful PDE inhibitors. However, the field is still evolving, and further research is needed to fully exploit the therapeutic potential of PDE inhibitors.

## Author contributions

**Conceptualization:** Mansour Alnazari, Abdulaziz Bakhsh, Sulaiman Abdullah, Saeed Al Qahtani, Walaa Borhan, Emad Rajih, Ahmed M. Alshehri.

**Methodology:** Mansour Alnazari, Abdulaziz Bakhsh, Sulaiman Abdullah, Saeed Al Qahtani, Walaa Borhan, Emad Rajih, Ahmed M. Alshehri.

**Writing – original draft:** Mansour Alnazari, Abdulaziz Bakhsh, Sulaiman Abdullah, Saeed Al Qahtani, Walaa Borhan, Emad Rajih, Ahmed M. Alshehri.

**Writing – review & editing:** Mansour Alnazari, Abdulaziz Bakhsh, Sulaiman Abdullah, Saeed Al Qahtani, Walaa Borhan, Emad Rajih, Ahmed M. Alshehri.
